# Evidence for P-Glycoprotein Involvement in Cell Volume Regulation Using Coulter Sizing in Flow Cytometry

**DOI:** 10.3390/ijms160714318

**Published:** 2015-06-24

**Authors:** Jennifer Pasquier, Damien Rioult, Nadine Abu-Kaoud, Jessica Hoarau-Véchot, Matthieu Marin, Frank Le Foll

**Affiliations:** 1 Stem Cell and Microenvironment Laboratory, Weill Cornell Medical College in Qatar, Education City, Qatar Foundation, Doha 24144, Qatar; E-Mails: naa2037@qatar-med.cornell.edu (N.A.-K.); jeh2036@qatar-med.cornell.edu (J.H.-V.); 2 Department of Genetic Medicine, Weill Cornell Medical College, New York, NY 10022, USA; 3 UMR_I 02 INERIS-URCA-ULH SEBIO Unité Stress Environnementaux et BIOsurveillance des milieux aquatiques, Université de Reims Champagne-Ardenne, BP-1039-Reims Cedex 2, 51687 Reims, France; E-Mail: damien.rioult@univ-reims.fr; 4 UGSF, UMR CNRS 8576, équipe Régulation des Signaux de Division, Université de Lille, Sciences et Technologies, 59009 Villeneuve d’Ascq, France; E-Mail: matthieu.marin@univ-lille1.fr; 5 Laboratory of Ecotoxicology UPRES EA 3222, IFRMP 23, University of Le Havre, 76058 Le Havre, France; E-Mail: frank.lefoll@univ-lehavre.fr

**Keywords:** multi-drug resistance, P-glycoprotein, cell volume, flow cytometer, MCF7

## Abstract

The regulation of cell volume is an essential function that is coupled to a variety of physiological processes such as receptor recycling, excitability and contraction, cell proliferation, migration, and programmed cell death. Under stress, cells undergo emergency swelling and respond to such a phenomenon with a regulatory volume decrease (RVD) where they release cellular ions, and other osmolytes as well as a concomitant loss of water. The link between P-glycoprotein, a transmembrane transporter, and cell volume regulation is controversial, and changes in cells volume are measured using microscopy or electrophysiology. For instance, by using the patch-clamp method, our team demonstrated that chloride currents activated in the RVD were more intense and rapid in a breast cancer cell line overexpressing the P-glycoprotein (P-gp). The Cell Lab Quanta SC is a flow cytometry system that simultaneously measures electronic volume, side scatter and three fluorescent colors; altogether this provides unsurpassed population resolution and accurate cell counting. Therefore, here we propose a novel method to follow cellular volume. By using the Coulter-type channel of the cytometer Cell Lab Quanta SC MPL (multi-platform loading), we demonstrated a role for the P-gp during different osmotic treatments, but also a differential activity of the P-gp through the cell cycle. Altogether, our data strongly suggests a role of P-gp in cell volume regulation.

## 1. Introduction

Each living cell type seems to have a form and volume that is well defined, and determined by the size of their cytoplasmic membrane as well as their cytosolic content. However, variations of osmotic pressures lead to cell volume regulation [1,2]. In the hypotonic or hypertonic context, the ability of cells to regulate their volume in a short period of time (around a minute) in order to avoid swelling or shrinkage is a fundamental mechanism [3]. Under hypo-osmotic conditions, cells are able to escape a burst after swelling by activation of a mechanism known as regulatory volume decrease (RVD) [4]. Interestingly, even under a regular osmotic pressure, the cell volume is fluctuating in response to cell events such as cell proliferation, migration, glycolysis or cell death [5,6,7,8].

P-glycoprotein (P-gp, *ABCB1*), a member of the ATP-binding cassette (ABC) superfamily of transporters, is a 170-kD transmembrane glycoprotein involved in the multi-drug resistance (MDR) phenotype [9,10]. The link between P-gp expression and cell volume regulation has been widely debated over the past decades [1,11,12]. Some studies reported that the P-gp is able to increase the magnitude of cell volume activated chloride currents, and so modulate the RVD [13,14,15,16]. On the contrary, other studies did not find any functional correlation between P-gp and volume regulation [17,18,19,20]. In 2005, our team demonstrated the role of P-gp in the regulation of volume activated chloride currents using wild-type human breast cancer MCF7 cells, and a doxorubicin-selected MDR variants [21]. Recently, it has been shown that apoptotic resistance in MDR P-gp overexpressing ascite tumour cells, involved impairment of the apoptotic volume decrease (AVD), an apoptotic event similar to the RVD [22]. Finally, in 2012, a team working on hepatocytes of the fish, *Sparus aurata*, suggested the involvement of the P-gp in the RVD [23].

The difference between all these studies could originate from the models and the methods used. Therefore, technological challenges still exist and the technique’s accuracy to measure both cell volume and P-gp activity/expression seems essential.

In this paper, we develop an original method based on flow cytometry analysis to study the role of the P-gp in the RVD in response to hypo-osmotic shocks. By using the Coulter-type channel of the cytometer Cell Lab Quanta SC MPL, we were able to follow accurately the electronic volume (EV) of the cells during either osmotic and/or pharmacologic treatment types.

## 2. Results

### 2.1. Resistant and Sensitive Variants Display Different Cell Volumes, Shapes and Membrane Capacitance

**Figure 1:**
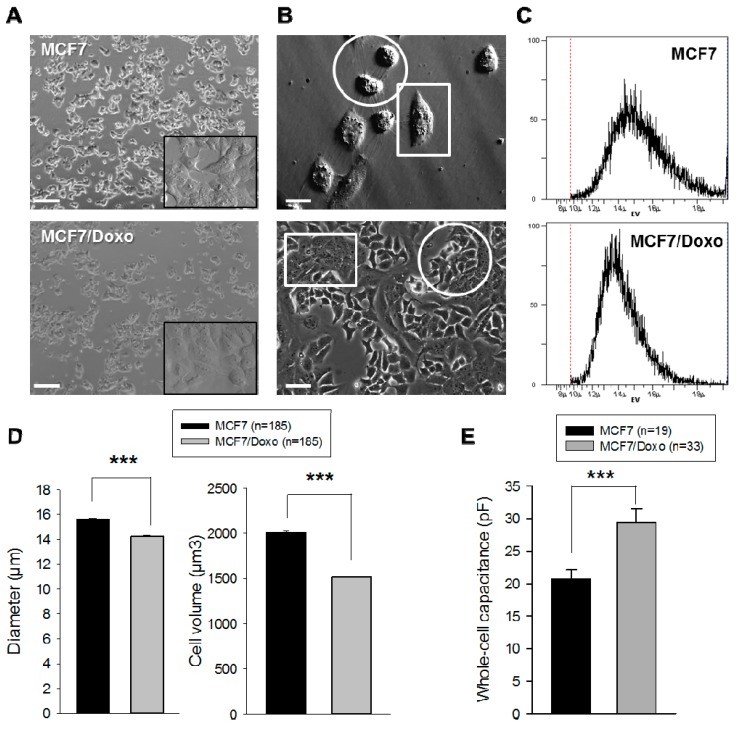

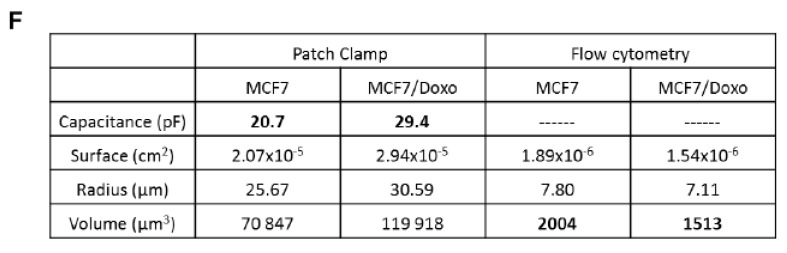
MCF7 and MCF7/Doxo display different morphological features. (**A**) Spatial organization of MCF7 and MCF7/Doxo in phase contrast microscopy. Scale bar: 50 µm; (**B**) Hoffman modulation contrast (**top**) and phase contrast micrographs (**bottom**) of MCF-7 and MCF-7/Doxo in co-cultures. Dishes were seeded with a 50:50 mixture of MCF-7:MCF7/Doxo at day zero. Morphological differences permit an immediate identification of each cell subpopulation islets. MCF7 appeared birefringent and round (rounds) whereas MCF-7/Doxo are more flat and spread (square). Scale bar: 20 µm; (**C**) Cell volume of MCF7 and MCF7/Doxo in flow cytometry. The electronic volume (EV) was determined by the flow cytometer according to the Coulter Principle; (**D**) Histograms giving the mean cell diameter (**left**) and volume (**right**) for 185 repeated experiments. The MCF7 appear significantly bigger than the MCF7/Doxo. Data are presented as mean ± SEM; (**E**) Whole-cell capacitance measurements of MCF7 and MCF7/Doxo. MCF7/Doxo display a higher whole-cell capacitance than the MCF7. Data are presented as mean ± SEM; (**F**) Summary of the different size measured or calculated for the MCF7 and MCF7/Doxo. *** *p <* 0.001*.*

Our group previously studied P-gp overexpression and activity in the MCF7/Doxo variant [24,25,26]; cell volumes of MCF7 and MCF7/Doxo were measured using different methods (Figure 1). In classical microscopy, morphological differences between the two variants could be verified (Figure 1A). The MCF7 appeared more birefringent and round, whereas the MCF7/Doxo were more flat and spread. Using Hoffman modulation contrast imaging on a freshly plated 50:50 co-culture, we revealed a clear morphological difference between MCF7 and MCF7/Doxo (Figure 1B top panel). After four days of co-culture, it was possible to observe a stable and unique spatial organization with the formation of MCF7 islets surrounded by MCF7/Doxo (Figure 1B bottom panel).

These differences have been confirmed using the Cell Lab Quanta MPL flow cytometer. This system exploits the Coulter principle for an accurate volume determination instead of the low-angle laser light scattering technique implemented in most of the cytometers. In short, as particles suspended in a saline solution are drawn through the small aperture of an insulated electrical sensor, they displace an equal volume of electrolyte solution that creates a resistance and leads to a voltage pulse. The voltage pulse intensity is proportional to the particle volume, thus called Electronic Volume (EV). Figure 1C displays the diameter (in µm) of the cells in suspension in a 300 mOsmol/kg H_2_O. A statistical analysis performed on more than 300 measurements revealed that the MCF7 were smaller in diameter and volume compared to the MCF7/Doxo (Figure 1D). It is also interesting to note that the MCF7 seemed to be less homogeneous in size than the MCF7/Doxo as revealed by the variation coefficient for the diameters (40.03% ± 0.05% and 30.85% ± 0.85%, respectively).

Finally, using electrophysiology, we measured the whole-cell capacitance of the two variants, 20.7 ± 1.4 pF (*n =* 19) for MCF7 and 29.4 ± 2.1 pF (*n =* 33) for MCF7/Doxo, respectively (Figure 1E). Considering a constant specific capacitance of C_S_ = 1 µF/cm^2^ [27] for the plasma membrane, these results indicate that the membrane electric surface is higher in the MCF7/Doxo compared to wild-type. This observation seems to contradict the one obtained by the volume Coulter (Figure 1F).

### 2.2. Cell Volume Monitoring during Hypo-Osmotic Shocks

**Figure 2:**
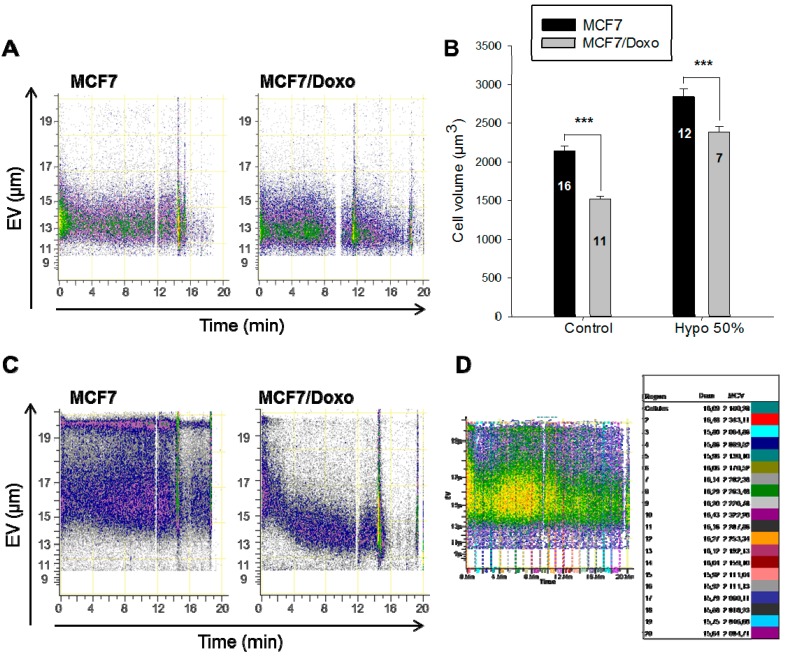

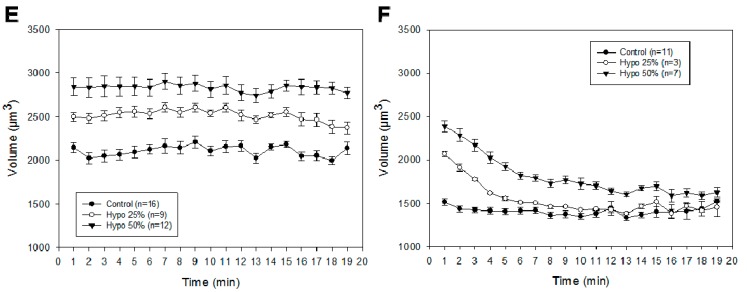
Cell volume monitoring during hypo-osmotic shocks. (**A**) Cell volume monitoring of MCF7 and MCF7/Doxo cells in normotonic conditions. The two graphs represent flow cytometry plots of the electronic volume (EV) *vs.* time, during the 20 min of the experiment; (**B**) Cell volumes after one minute of hypo-osmotic stress. Cell volumes were recorded by flow cytrometry before (control) or after one minute of a 50% hypo-osmotic shock (Hypo 50%). Data are presented as mean ± SEM; (**C**) Cell volumes monitoring in MCF7 and MCF7/Doxo cells during a hypo-osmotic shocks. The two flow cytometry plots represent the electronic volume (EV) of the cells subjected to a 50% hypo-osmotic stress during the 20 min. MCF7 cells (**left** plot) increased their volume without any compensation phenomenon. After a significant volume increase, MCF7/Doxo cells (**right** plot) retrieved their original volume; (**D**) Data extraction method. Nineteen equal gates of 1 min length have been created and applied to all samples. The mean cell diameter (Diam.) and of the mean cell volume (MCV) in each region have been extracted with the Cell Lab Quanta analysis software; (**E**) Cell volume monitoring of MCF7 under different conditions. Cell volumes of MCF7 have been recorded by flow cytometry in isotonic conditions (control) or during 25% or a 50% hypo-osmotic challenges; (**F**) Cell volume monitoring of MCF7/Doxo cells under different conditions. Cell volumes of MCF7 have been recorded by flow cytometry in isotonic conditions (control) or during 25% or a 50% hypo-osmotic challenges. *** *p <* 0.001.

Flow cytometry coupled to Coulter EV measurements represents a valuable approach to monitor cell size variations in real-time. Thus, we have used this possibility to carry out analysis of volume change time-course of cells undergoing osmotic challenges in suspension at a low flow rate (25 µL/min). With these settings, the cell volume distributions can be determined over 20 min. This approach is better than the traditional volume coulter method that allows only a static measurement of the cell volumes. As shown in Figure 2A, cell volumes of the two variants were stable over the 20 min period. However, during 50% hypo-osmotic shocks (150 mOsmol/kg H_2_O), a significant swelling of both variants was detected two minutes after the substitution of the isotonic solution with the hypotonic one (Figure 2B). The temporal monitoring of the volume compensation, RVD, revealed important differences between the two variants (Figure 2C). While the MCF7/Doxo cells were able to compensate the swelling drove by hypotonicity, the MCF7 cells could not. For the MCF7/Doxo cells, cell volume normalization appeared after less than 10 min, whilst no RVD mechanism was noticed after 20 min for the MCF7 cells.

This experiment has been repeated several times in isotonic and hypotonic conditions. To analyze the large number of points generated (200,000 cells analyzed/experiment), 20 successive gates of 1-min cell volume continuous recording have been set (Figure 2D). For each 1-min interval, the mean cell volume has been determined. In these conditions, it has been possible to draw a graph representative of the different experiments (Figure 2E,F). After 20 min, the MCF7 were not able to display any RVD mechanism, neither under 25 nor under 50% hypo-osmotic shocks (Figure 2E). On the contrary, in the MCF7/Doxo cells, a RVD was set up immediately and re-established normotonic volume values after 7 min in both 25% and 50% hypo-osmotic shocks (Figure 2F).

### 2.3. RVD in MCF7/Doxo Cells Is Dependent on P-gp Activity

**Figure 3:**
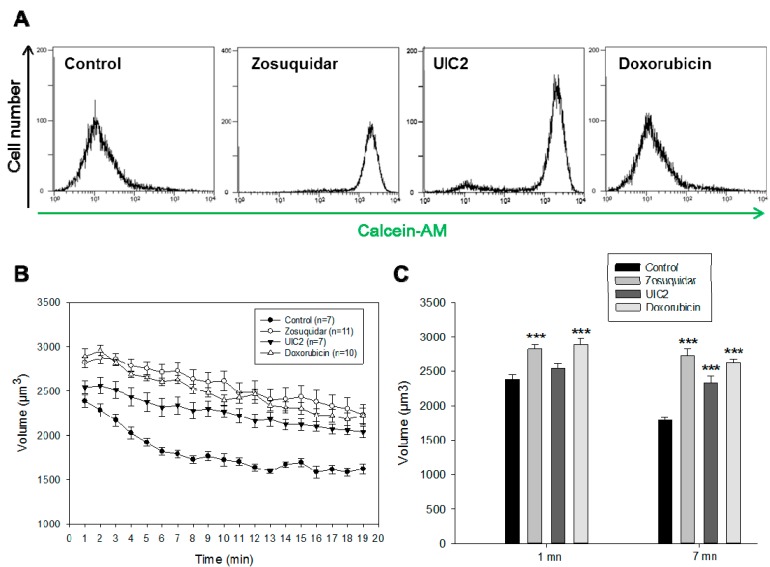

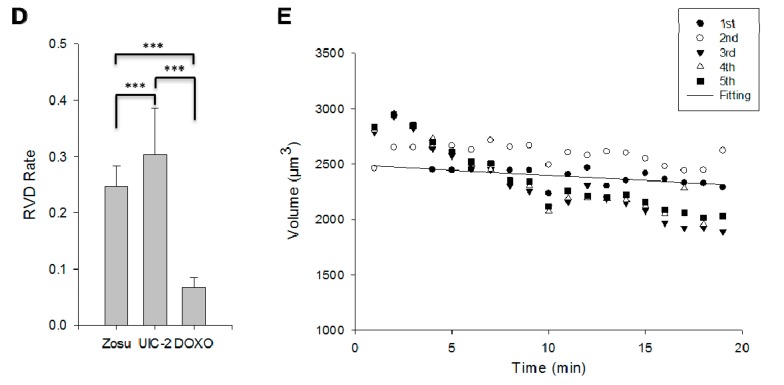
RVD in MCF7/Doxo is dependent on the P-gp activity. (**A**) P-gp activity. P-gp activity was followed with calcein-AM as a fluorescent probe. In each flow cytometry measurement, a sample of 10,000 cells was analyzed. Superimposed all-events histograms of calcein fluorescence distribution (log scale) in control MCF-7/Doxo cells (**left** plot) and MCF-7/Doxo pre-incubated with the P-gp antagonist zosuquidar and UIC2 (10 μM, **middle** plots) or with doxorubicin (**right** histogram); (**B**) Cell volume monitoring of MCF7/Doxo in presence of P-gp modulators; (**C**) The histogram represents the mean of the cell volume after 1 or 7 min of 50% hypo-osmotic stress for the experiments presented in **B**. While under the control conditions (black) the cells retrieved their original volume after 7 min, the cells in presence of the P-gp modulators were not able to compensate their volume increase. Data are presented as mean ± SEM, *** *p <* 0.001.; (**D**) RVD rate of MCF7/Doxo cells in 50% hypo-osmotic shock (*** *p <* 0.001); and (**E**) Graphs show MCF7/Doxo cell volumes from five independent samples analyzed by flow cytometry after 50% hypo-osmotic conditions in the presence of doxorubicin. The data were fitted with Sigma Plot.

The role of efflux activity of P-gp in the regulation of the RVD has been investigated using different ligands of P-gp chosen for their different mechanisms of action. Thus, a non-competitive inhibitor, zosuquidar (20 µM), a conformational monoclonal antibody, UIC2, and a P-gp substrate used in chemotherapy treatment of breast cancer, doxorubicin (20 µM), have been used. First we quantified the effect of each compound on the P-gp efflux activity with the calcein-AM as a fluorescent allocrite probe (Figure 3A). Cells expressing high levels of P-gp rapidly extrude nonfluorescent calcein-AM from the plasma membrane. As a result, fluorescence intensity is inversely related to P-gp [26]. The conformational antibody UIC2 and zosuquidar completely abolished the P-gp activity in MCF7/Doxo cells, while doxorubicin did not alter the ability of MCF7/Doxo to expel calcein. In isotonic conditions, the cell volumes of MCF7/Doxo were not modified by any of the three ligands (data not shown). On the contrary, after 50% hypo-osmotic shocks, RVD was inhibited by the 3 P-gp modulators (Figure 3B). Analysis at 1 min shows clearly that the untreated MCF7/Doxo cells were already engaged in a RVD process while, in the presence of zosuquidar or doxorubicin, they did not even reach their maximal volume (Figure 3C). After 7 min, the three ligands were able to significantly decrease RVD kinetics in MCF7/Doxo. Comparisons of the RVD rates revealed that doxorubicin slowed down most of the RVD process (Figure 3D). The experimental points of five different experiments, under doxorubicin treatment, were fitted with straight lines (Figure 3E). The small slope demonstrated the nearly total inhibition of the RVD in the presence of doxorubicin.

### 2.4. P-gp Activity and Cell Cycle

**Figure 4:**
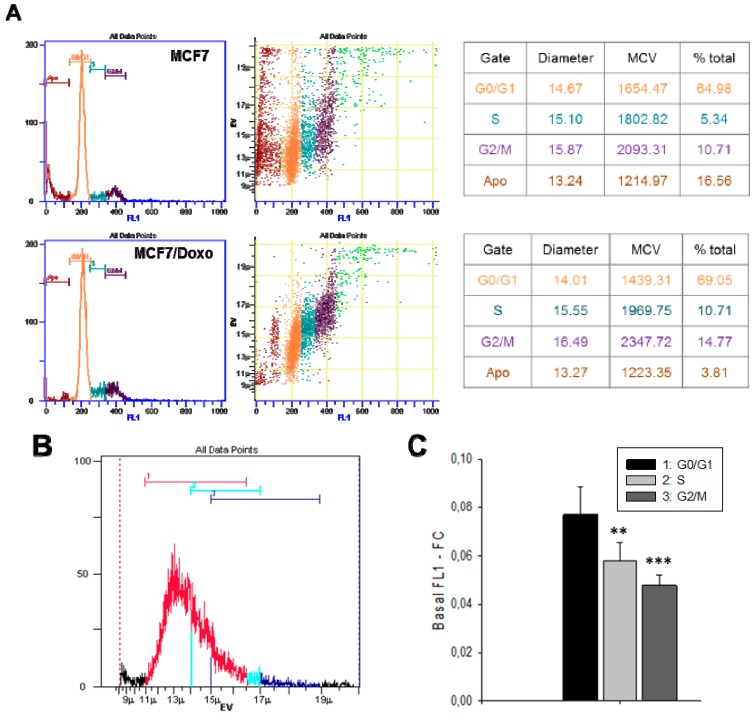

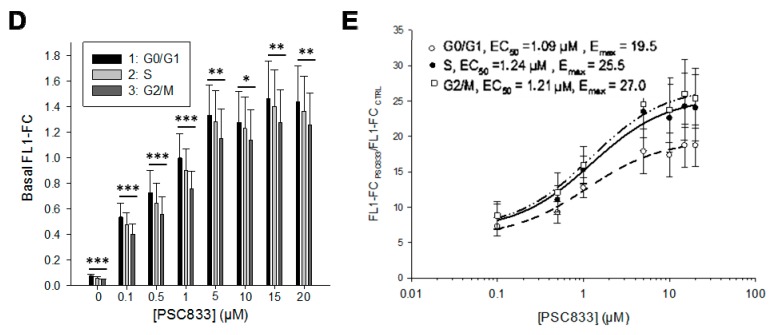
MCF-7/Doxo cells exhibit a cell cycle-dependent P-gp activity. (**A**) Cell cycle of MCF7 and MCF7/Doxo was determined by DNA content measurements with the nucleic acid dye Hoechst 33342. Four gates corresponding to hypoploid particles (Apo), cells in G0/G1, cells S or cells G2/M phases were defined based on the FL1 level (**left** plots) and applied on the cell volume EV (**right** plots). The mean cell volume of the MCF7 and MCF7/Doxo were extracted from each gate (tables); (**B**) The gates defined in A on the FL1 parameter were switched on the EV channel to be applied to the following experiments; (**C**) P-gp activity within the cell cycle. The P-gp activity was assayed with calcein-AM and the amount of fluorescence per cell was expressed as FL1-FC (fluorescent light in channel 1-fluorescence concentration) which is the fluorescent light (FL) divided by the electronic volume (EV) determined by the flow cytometer according to the Coulter Principle; (**D**) P-gp inhibition with PSC833 within the cell cycle. Different concentrations of PSC833 were used and the P-gp activity was assayed with calcein-AM. The histogram represents the FL1-FC mean in each gate. The stars represent the difference between the three groups after an ANOVA test; and (**E**) PSC833 dose response curves expressed as mean FL1-FC. Each point represents mean ± SEM (10 independent experiments) of FL1-FC, expressed as the ratio of signals in the presence of a blocker to signal in control conditions in each gate. Four-parameter logistic dose-response curves were fitted to the data to obtain blocker potencies (half-maximal effective concentration, EC_50_) and efficacies (maximum response, *E*_max_). Data are presented as mean ± SEM with *n* = 10 independent assays per data point. * *p <* 0.05, ** *p <* 0.01, *** *p <* 0.001.

In isotonic conditions, cell proliferation is one of the major events leading to cell size regulation. Thus, in the present work, cell volume changes were analyzed for the two variants during the cell cycle (Figure 4A). Measurements of large cell populations indicate that MCF7 exhibits larger size than MCF7/Doxo cells. In phase S or G2/M phases however, the volume of MCF7/Doxo was found to be higher than that of MCF7. In order to study the P-gp activity during the cell cycle, we developed a gating strategy based on the cell volumes defined by the cell cycle analysis (Figure 4B). However, cell size variations affect fluorescence signals and induce alteration in cell calcein content assessments. To overcome these potential variations, we have used the FL1-FC parameter of the cytometer, in which fluorescence intensity (FL1) was normalized as a ratio of fluorescence concentration to an accurate cell sizing determined by Coulter-type electronic volume (EV) [26]. Results indicate that the basal fluorescence of the calcein was more important in gate 1 (corresponding to the cells in G0/G1), which correlates with a lower P-gp activity (Figure 4C). The best P-gp activity was detected in cells in G2/M which are the cells displaying the larger size. After this, we used a non-competitive P-gp inhibitor, the PSC833, and tested its efficacy on the calcein accumulation at the different stages of the cell cycle (Figure 4D). Dose response curves represented in Figure 4E allowed us to determine EC_50_ (half maximal effective concentration) and *E*_max_ (maximal effect) values of PSC833 for each gate. PSC833 maximal effect was increasing with the progression of the cells in the cell cycle, without any modifications of potency (EC_50_).

## 3. Discussion

The role of P-gp in cell volume regulation has been discussed widely over the past 20 years. In the present study, we used an original, non-intrusive and label-free method based on flow cytometry coupled to Coulter volume determination to compare RVD in wild-type and doxorubicin-resistant MCF7, during hypo-osmotic challenges. This technique allowed us to follow not only the volume variation of cells in a live scenario, but also to concurrently study cell volumes and P-gp activity.

First, we demonstrated that MCF7 cells do not respond as MCF7/Doxo cells to hypo-osmotic shocks. In fact, while MCF7 cells underwent a persisting volume increase throughout the 20 min of the experiment, MCF7/Doxo cells were able to offset osmosis and to cancel swelling in less than ten minutes, suggesting strongly that the P-gp is promoting RVD. Historically, because of sequence similarities with an ABC transporter functioning as a chloride channel (the cystic fibrosis transmembrane regulator, CFTR), a role for P-gp in volume regulation has been investigated and, as a matter of fact, confirmed in P-gp overexpressing cells [13,28]. Thus, in 1992, Gill *et al.* stated that P-gp could form a channel itself, or a component of such a channel [28]. Two years later, Sadini and collaborators demonstrated in *mdr1/ABCB1* transfected NIH-3T3 cells that short-term hypotonic conditions caused an inhibition of P-gp activity [29]. In 1998, using a drug-sensitive cell line (MCF-7) and a P-gp-expressing derivative (BC19/3), the authors demonstrated an increase in the magnitude of cell volume activated chloride currents in BC19/3 cells, but ruled out the possibility of P-gp being itself the channel responsible for the volume-activated currents [14]. Conversely, other studies concluded that RVD and osmoregulatory chloride currents were not related to P-gp expression in resistant cell lines or in cells transfected with *mdr1/ABCB1* transcripts [17,19,20,30] or injected with the protein [31]. To add to the confusion, in 2002, Chen *et al.* abolished volume-activated chloride currents in bovine pigmented ciliary epithelial cells by using *mdr1/ABCB1* antisense oligonucleotides; suggesting their dependence on endogenous P-gp expression [32]. In 2005, using the same cell lines as in the present study, our team demonstrated that during hypotonic challenges, swelling-activated chloride currents were significantly activated faster and with larger densities in MCF7/Doxo cells than in MCF7 cells [21]. We also demonstrated the inhibition of this current by P-gp ligands, including conventional substrates such as doxorubicin, as well as antibodies. These results are in accordance with the total inhibition of RVD obtained in the presence of doxorubicin herein. In addition, our study is the first that combines non-invasive measurements of the cell volume and a cell model overexpressing the P-gp only by selection and not by transfection or injection.

More recently, in 2014, a relation between P-gp, AQP5 and drug resistance has been established [33]. By using AQP5-siRNA to silence AQP5 in the colon-derived cell line HT-29, the authors obtained a decrease of P-gp expression as well as of other actors of drug resistance, such as GST-π, and TOPO II. These findings are of prime importance since aquaporins are known to be involved in the early phase of apoptosis, characterized by a cell shrinkage, named apoptotic volume decrease (AVD) [34]. For instance, the under-expression of aquaporin AQP8 and 9, in hepatocellular carcinoma, is responsible for the resistance to starvation or TGFβ-induced apoptosis [35]. Resistance to apoptosis is one of the principal features of tumor cells [22,36,37], hence we could hypothesize a role of P-gp in AVD-impairment as a mechanism associated with death evasion. In the presence of cytotoxins such as doxorubicin, and while pumping the substrate out of the cells, the P-gp could be involved in pathways counteracting cell shrinkage and may thus contribute to avoid apoptosis. Alteration of AVD could therefore be added to the classical mechanisms of resistance that the cancer cells use against chemotherapeutic agents.

In parallel to the mechanisms of cell volume regulation in hypotonic conditions, we demonstrated obvious differences in term of morphology and cell volume between the two variants, in regular osmotic pressures. MCF7 cells in suspension in an isotonic solution have an average volume of about 2000 μm^3^, while MCF7/Doxo cells have a much smaller volume of 1500 μm^3^. This is in accordance with the results of Yang and collaborators demonstrating that human ovarian cancer cell line SKOV3 that started to express P-gp after a selection with cisplatin, exhibited dramatic changes in morphology, including reduction in cell size, loss of cellular projections and clustering [38]. In ascites tumor cell lines, a high correlation between the ability to pump out daunorubicin (by the P-gp), and the decrease in cell volume detected has also been demonstrated in resistant cell lines [39].

**Figure 5:**
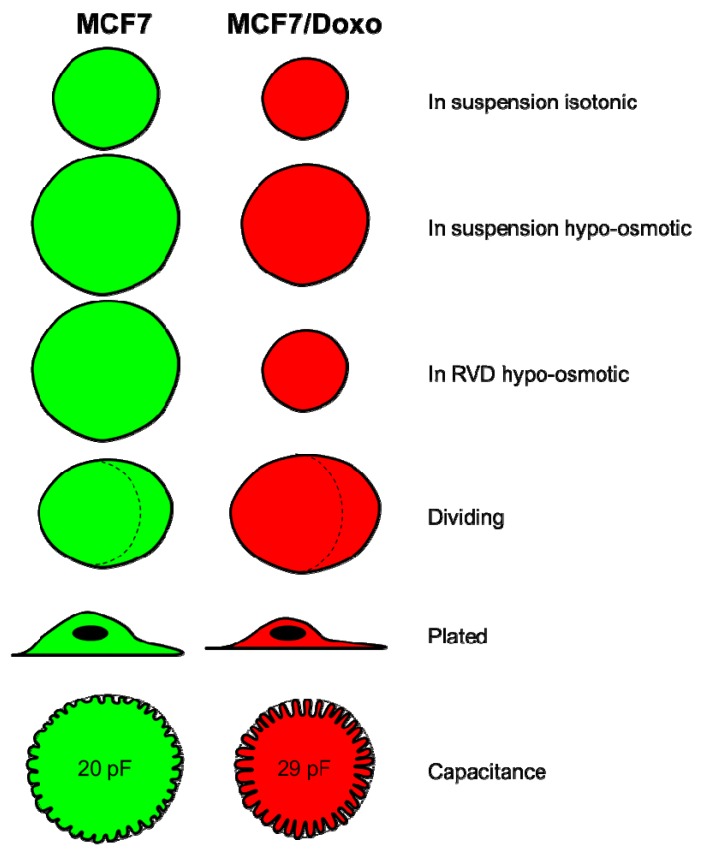
Schematic representation of MCF7 and MCF7/Doxo cells in each situation.

The measurement of the membrane capacitance in the whole cell configuration of the patch clamp technique revealed that the enclosed volume of the membrane is theoretically 70,847 μm^3^ for MCF7 cells and 119,918 μm^3^ for MCF7/Doxo cells. This technique gives some information on the total area of the plasma membrane. Therefore, although the difference between the values obtained by Coulter volume and volume per surface area may seem significant, the folding structure of the membrane, which indeed contributes to the membrane capacity and not to the volume, could be an explanation. Thus, the measurements of the membrane capacitance suggest that the two variants have a different membrane conformation, whereby MCF-7/Doxo cells contain more membrane folding than MCF-7 cells (Figure 5). The volume differences between the two variants could also be explained by the difference in terms of morphology in phase contrast on adherent cells, since it was mentioned that MCF7 cells are more birefringent, so more swollen than MCF7/Doxo cells. We can hypothesize that through peculiar interactions with lipids and favorable locations in the plasma membrane, P-gp can influence the membrane structure [40,41,42].

Recently, many authors raised the possibility of a G1/S volume checkpoint controlling the progression through the cell cycle [43,44]. Even if the concept of a cell size checkpoint in the cell cycle has been well established in yeast and other organisms [45], it yet remains controversial in mammalian cells [46,47,48]. Some authors suggest that translational checkpoints at the G1/S transition could set a lower cell size limit [49,50,51], while others claim the importance of an upper limit of cell volume in progression through M phase [52,53]. Even if the cell volume regulation seems mandatory for the cell cycle progression and cell division, it remains unclear if these events are triggering the cell cycle itself or if they are just actors activated through the regular cyclin dependent kinase/cyclin (Cdk/cyclin) complex [43,44]. As per our results reported here, it seems that MCF7 cells are able to undergo a complete normal mitosis. Moreover, in a previous publication, we confirmed that there is no difference between the proliferation capacity of MCF7 and MCF7/Doxo, except the resistance to chemotherapy drugs exhibited by MCF7/Doxo cells [25]. Therefore, even if during the 20 min of hypotonic stress MCF7 cells were not able to display any efficient volume compensation, it seems that they are completely able to activate volume regulatory mechanisms in the cell cycle context.

Finally, we were also able to show that the cells involved in mitosis display the highest P-gp activity. During mitosis, cells are subjected to constant volume variation that may reinforce the role of P-gp in cell volume regulation. Moreover, many drugs used in chemotherapy target cell division, and a high expression level of P-gp has been linked to resistance to these drugs [54,55]. We can thus suggest a new mechanism of resistance to this drug, independent of the drug efflux abilities of P-gp; this data therefore allows us to imagine the existence of new mechanistic consequences of P-gp overexpression.

## 4. Material and Methods

### 4.1. Cell Cultures

The study was carried out with human breast carcinoma derived cells, MCF-7 cells and its multi-drug resistant variant (MCF-7/Doxo); kindly provided by J.-P. Marie (Hôtel Dieu, Paris, France). MCF7/Doxo cells were isolated by stepwise selection with increasing concentrations of doxorubicin [56]. Cells were maintained in RPMI 1640 (Sigma, St. Louis, MO, USA) containing 5% of heat-inactivated fetal bovine serum (Sigma), 2 mM l-glutamine (Sigma, St. Louis, MO, USA), 1% antibiotic/antimycotic solution (Sigma), and incubated in a humidified atmosphere containing 5% of CO_2_ at 37 °C. Doxorubicin (1 μM) was added to the culture medium for the maintenance of the multi-drug resistant phenotype of MCF7/Doxo cells.

### 4.2. Reagents

All reagents were of the highest grade of purity and quality available. Purified doxorubicin (DOXO), DMSO, and phosphate buffer saline (PBS buffer, pH 7.4) were purchased from Sigma. Calcein acetoxy-methylester (calcéine-AM) was supplied by Invitrogen Life Technologies (Carlsbad, CA, USA). SDZ PSC833 and zosuquidar were kindly provided by J.-P. Marie (Hôtel Dieu, Paris, France). The final concentration of DMSO and H_2_O was less than 0.1%.

### 4.3. Flow Cytometry

For P-gp expression, activity or cell cycle, the fluorescent light (FL) was quantified using a Cell Lab Quanta SC MPL flow cytometer (Beckman Coulter, Fullerton, CA, USA) equipped with a 22 mW 488 nm excitation laser. Voltage settings of photomultipliers were not modified throughout the experiments [24,25]. Each analysis consisted of a 10,000 events record, triggered on electronic volume (EV) as primary parameter, according to a particle diameter exceeding 8 µm.

For the volume monitoring, the cytometer was operated at a flow rate of 25 µL/min. Cell sizes were accurately determined using the Coulter-type electronic volume (EV) channel of the cytometer, after calibration with 10-μm FlowCheck microspheres [57]. The cell volume distribution was measured for 20 min.

### 4.4. Osmostic Challenges and RVD Rate Calculation

Osmolality of solutions was checked and adjusted by using a Wescor Vapro 5520 vapor pressure osmometer (Wescor Elitech, Logan, UT, USA). RVD rate is the time constant. It has been calculated using the Software Sigma Plot 11 (Systat Software Inc., Chicago, IL, USA). Data have been fitted with the Regression wizard using the Exponential Decay function. There are three parameters and the equation is y = y0 + a × e(−bx), where “b” is the decrease constant corresponding to the time constant represented in the bar graph (Figure 3D).

### 4.5. P-gp Activity

The calcein-AM efflux assay was used as previously described [26]. Briefly, resuspended cells were loaded with 0.25 µM calcein acetoxy-methylester (Invitrogen) in RPMI for 15 min at 37 °C in the dark. Green FL was quantified via the FL1 channel (log scale) through a 525 nm band pass filter.

### 4.6. Cell Cycle

By using a cell-penetrating DNA-binding dye, Hoechst 33342, cellular DNA content could be measured without fixation and permeabilization. Briefly, cells were incubated with 20 μg/mL Hoechst 33342 in cell culture medium for 45 min at 37 °C. The stained cells were analyzed by the Quanta SC MPL using the Hg arc lamp with a 355/37BP excitation filter as excitation and detected on the FL1 with a 465/30BP filter. Data acquisition was carried out by triggering on FL1.

### 4.7. Electrophysiology

For patch-clamp recordings, cells were allowed to attach for 3–4 h to 20 mm diameter glass cover slips. The electrophysiological studies were performed at room temperature (23–25 °C) on the stage of an inverted microscope (Nikon TE2000, Champigny-sur-Marne, France) using the whole-cell configuration of the patch-clamp technique in the voltage-clamp mode. Patch pipettes were pulled from borosilicate glass capillaries (Harvard Apparatus, Holliston, MA, USA) with a P-97 horizontal puller (Sutter Instrument, Novato, CA, USA). The intracellular (pipette) solution contained (in mM): 134 CsCl, 1.8 CaCl_2_, 1 MgCl_2_, 2 ethylene glycol-*bis*(b-aminoethylether)-*N*,*N*,*N'*,*N'*,-tetraacetic acid (EGTA), 10 4-(2-hydroxyethyl)piperazine-1-ethanesulfonic acid (HEPES) and 3 Na2ATP; adjusted to pH 7.2 with CsOH. The 20%-hypotonic bath solution (240 mOsmol/kg H_2_O) contained (in mM): 2.8 tetraethyl ammonium (TEA)-Cl, 100 *N*-methyl-d-glucamine (NMDG)-Cl, 2 MgCl_2_, 1 CoCl_2_ and 10 HEPES, adjusted to pH 7.4 with HCl. In order to avoid any change in ECl, the osmolality was set at 300 mOsM by adding mannitol. When in the bath and filled with the intracellular solution, patch pipettes had a resistance between 2 and 4 MΩ.

Recordings were made with an Axopatch 200B patch-clamp amplifier interfaced to a 1.5 GHz computer via a Digidata 1322 and pClamp 8.0 software (Axon instruments, Foster City, CA, USA). Cells with access resistances exceeding 10 MΩ immediately after gaining the whole cell configuration or during hypotonic challenge were discarded.

### 4.8. Statistical Analysis

All quantitative data were expressed as mean ± standard error of the mean (SEM). Statistical analyses were performed with SigmaPlot 11 (Systat Software Inc., Chicago, IL, USA). A Shapiro-Wilk normality test, with a *p* = 0.05 rejection value, was used to test normal distribution of data prior further analysis. All pairwise multiple comparisons were performed by one way ANOVA followed by Holm-Sidak posthoc tests for data with normal distribution or by Kruskal-Wallis analysis of variance on ranks followed by Tukey posthoc tests, in case of failed normality test. Paired comparisons were performed by Student’s *t*-tests or by Mann-Whitney rank sum tests in case of unequal variance or failed normality test. Statistical significance was accepted for *p* < 0.05 (*), *p* < 0.01 (**) or *p* < 0.001 (***). All experiments were performed in triplicate.

For the purpose of IC_50_ value calculation (dose-response curves of calcein-AM assay), data were fitted to a sigmoidal three parameters dose-response model [58]:

y = b + (a − b)/(1 + 10 (log IC_50_ − x))

where (y) is response, *i.e.*, the ratio of mean FL1-FC in the presence of PSC833 to mean FL1-FC in control condition, (b) represents minimum of response, (a) represents maximum of response Emax, (x) is logarithm of PSC833 concentration and IC_50_ (or EC_50_) is the concentration of inhibitor that corresponds to 50% of maximal effect.

## 5. Conclusions

In this study, we show evidence for a role of P-gp in cell volume regulation by combining microscopic observations, coulter volume, flow cytometry and electrophysiology. Taken together, our results strongly suggest that overexpression of P-gp in MCF7/Doxo cells has an impact on cell volume regulation in regular osmotic pressure and also during hypo-osmotic stresses. We highlight unexpected differences between two MCF-7 variants in regular osmotic conditions. Under osmotic challenge, P-gp overexpression influences cell volume regulation; it can then be supposed that P-gp overexpression contributes to chemoresistance not only by drug efflux, but also by volume stabilization and AVD-impairment. However, it should be emphasized that even if hypo-osmotic challenges associated with RVD monitoring remains an experimental approach to model AVD, a direct study of AVD seems necessary to validate our assumption. Overall, by multimodal cell volume monitoring, we were able to rekindle discussion on the role of P-gp in cell volume regulation.

## Abbreviations

ABC: ATP-binding cassette
AVD: apoptotic volume decrease
EV: electronic volume
MDR: MultiDrug Resistance
P-gp: P-glycoprotein
RVD: regulatory volume decrease
RVI: regulatory volume increase
